# Impact of extremely low frequency electromagnetic fields exposure on sleep quality and mental health in a Tunisian power plant: a cross-sectional study

**DOI:** 10.3389/fpsyt.2026.1755918

**Published:** 2026-04-24

**Authors:** Imène Kacem, Imen Jammeli, Chaima Sridi, Asma Gaddour, Manel Makhloufi, Asma Aloui, Asma Chouchane, Olfa El Maalel, Mohamed Kahloul, Nejib Mrizak

**Affiliations:** 1 Faculty of Medicine of Sousse, University of Sousse, Sousse, Tunisia; 2 Occupational Medicine Department, Farhat Hached University Hospital, Sousse, Tunisia; 3 Farhat Hached University Hospital, Laboratoire de Recherche (LR19SP03: Etude des risques et perspectives de prévention des maladies non transmissibles en milieu professionnel), Sousse, Tunisia; 4 Occupational Medicine Department, Ibn Jazzar University Hospital, Kairouan, Tunisia; 5 Occupational Medicine Department, Sahloul University Hospital, Sousse, Tunisia; 6 Anesthesia and Intensive Care Department, Sahloul University Hospital, Sousse, Tunisia

**Keywords:** anxiety, depression, extremely low-frequency electromagnetic fields, sleep disorders, stress

## Abstract

**Introduction:**

Extremely low-frequency electromagnetic fields (ELF-EMFs) are ubiquitous in our daily life. They may have an impact not only on physical health but also on mental health.

**Objectives:**

To assess the impact of occupational exposure to the ELF-EMFs on sleep quality, depression, anxiety and stress among workers at the Tunisian Electricity and Gas Company (TEGC).

**Methods:**

This was a cross-sectional study. The study population included two groups: an exposed group (EG), consisting of power plant employees, and a non-exposed group (NEG), consisting of administrative workers. Exposure to ELF-EMFs was assessed via spot measurements using a magnetometer. Sleep quality, depression, anxiety and stress were assessed by the French versions of the Pittsburgh Sleep Quality Index (PSQI) and the Depression, Anxiety and Stress Scale (DASS-21).

**Results:**

Seventy-seven participants in the EG and 88 participants in the NEG were included in the study. The median value of the ELF-EMFs was 5.86 μT at the power plant [min 0.1, max 40.34 μT]. According to the PSQI global score, 64.9% of the EG had poor sleep quality versus 29.5% of the NEG. Depression was registered in 24.7% of EG and in 3.4% of NEG. Anxiety was noted in 23.4% of the EG and in none of the NEG. Stress was found in 46.8% of the EG and none of the NEG. After multivariate analysis, ELF-EMF exposure was significantly associated with poor sleep quality and depression.

**Conclusion:**

The present study revealed that ELF-EMFs can affect sleep and mental health. Further studies are needed to explain the mechanism involved.

## Highlights

- What is already known on this topic: Extremely low-frequency magnetic fields (ELF-EMFs) are ubiquitous in daily life and in occupational environments. There are only a few studies that have assessed the impact of these types of fields on mental health especially quality of sleep, depression, anxiety and stress.
- What this study adds: The results of this study have revealed that occupational exposure to ELF-EMFs among workers in a power plant can lead to depression and poor sleep quality.
- How this study might affect research, practice or policy – This study highlights the importance of assessing the mental health of workers in a power plant particularly those who are exposed to EMFs.

## Introduction

Extremely low-frequency electromagnetic fields (ELF-EMFs) such as vibrations, noise, heat and optical radiation are ubiquitous in our daily lives, but are not always directly perceived by the human senses. Exposure to these types of fields may arise from natural sources including the Earth’s magnetic field and electrophysiological signals produced by living organisms. They can also be artificially generated by power lines, televisions and household appliances (1, 2).

According to the World Health Organization (WHO), ELF-EMFs are all forms of non-ionizing radiation with a frequency ranging from 0 to 300 Hz (1, 3). ELF-EMFs are created by the production of electricity, transmission over power lines, transformers and by the network distribution. Power plant workers are particularly exposed to ELF-EMFs and their associated health impacts (4, 5).

For many years, public opinion has focused on the impact of ELF-EMF exposure on physical and mental health (6). Many studies have examined the effects of this type of fields in both daily life and occupational settings. Wertheimer (1979) was the first to explore the association between exposure to electromagnetic fields and childhood leukemia. Since then, an increasing amount of research has suggested an association between exposure to ELF-EMFs and the incidence of various types of cancer, including childhood leukemia (7, 8). Since 2002, these fields have been classified as a Group 2B carcinogen by the International Agency for Research on Cancer (IARC) for childhood leukemia, for residential exposure exceeding 0.4µT (9).

In addition, other effects have been suggested by *in vivo* and *in vitro* studies. Some authors have reported that exposure to ELF-EMFs may disrupt neuroendocrine activity and lead to stress, depression, anxiety, learning difficulties, sleep disorders and mental health alteration (5, 10, 11).

Several animal studies have investigated the repercussions of exposure to ELF-EMFs on the function of the central nervous system. These studies showed that ELF-EMFs can modulate the activity of the hippocampus, particularly cell proliferation and neurogenesis. As a result, the learning process and memory can be compromised (12, 13). Chronic occupational exposure to ELF-EMFs can also increase the risk of sleep disorders, depression and anxiety (11).

In a cross-sectional study carried out in China among 854 participants working in an electric power plant, the results showed that the daily occupational exposure to EMFs was positively associated with poor sleep quality (14). In another cross-sectional study conducted among electronic equipment repairers, the results showed that workers who were exposed to ELF-EMFs daily were more likely to have sleep insufficiency than the controls (15).

However, data available on this issue remain limited and controversial.

The present study aimed to assess the impact of chronic exposure to ELF-EMFs on sleep quality, stress, depression and anxiety among workers in a Tunisian power plant in Sousse.

## Methods

### Ethics statement

The study was conducted in accordance with the ethical principles of the Declaration of Helsinki. Ethical approval was obtained from the Ethics Committee of Sahloul University Hospital, Sousse, Tunisia (Ref: HS 02-2022; Date: 10/01/2022). All the participants received a clear verbal explanation of the study’s purpose, procedures, risks, benefits, and right to withdraw at any time. Verbal informed consent was obtained before data collection. The data were collected anonymously to ensure confidentiality.

### Study design

This is a cross-sectional, analytical study which enrolled workers in a Tunisian power plant called TEGC located in Sousse. This study was conducted over a six-month period, from June 1 to December 31, 2022.

### Participants and sampling

The study population was composed of two groups according to the level of exposure to ELF-EMFs. The exposed group (EG) was represented by workers of units B, C, and D of the power plant. The workers in these units were exposed to several sources of high ELF-EMFs, such as power generators, the boilers feeder pumps, circulating pump and electrical buildings. The non-exposed group (NEG) was represented by workers of the same company working in the administrative sections from other locations of the same company in Sousse, Tunisia (District North Sousse, District Sousse town center, District Enfidha and District Msaken).

Inclusion criteria were male workers who had been continuously employed, in one or more of the targeted regions of the company, for at least two years. The workers excluded from the study were those who used antioxidant supplements or medication that interferes with sleep; those who had a psychiatric history including depression, anxiety and sleep disorders before being employed; those who were involved in night work or shift work; and those who did not consent to participate.

In total, 77 power plant workers were included in the EG, and 88 workers were included in the NEG.

### Study participant and public involvement

In this study, neither the participants nor the public were involved in the design, conduct, reporting or dissemination plans of the research.

### Data collection

Data were collected via a pre-established, anonymous questionnaire and concurrent EMFs measurements. The questionnaire assessed sociodemographic and occupational characteristics, lifestyle habits, personal and family medical history, sleep quality, depression, anxiety, and stress levels. Following a detailed explanation of the study objectives and the acquisition of informed consent, the questionnaire was administered by the principal investigator.

### Operational variables

Sleep quality was assessed via the French version of the Pittsburgh Sleep Quality Index Questionnaire (PSQI). This self-administered questionnaire was developed by Buysse et al. (1989) to assess the sleep quality during the previous month (16). It is composed of 19 self-assessment questions that can be grouped into seven components: subjective sleep quality, sleep latency, sleep duration, habitual sleep efficiency, sleep disturbances, use of sleeping medication, and daytime dysfunction. Each item is scored from 0 to 3, and the sum of the seven components yields a PSQI global score ranging from 0 to 21 (17). The current study used the French version, which has been validated by Blais et al. (1997), showing acceptable levels of internal homogeneity, consistency and validity (18).

A PSQI global score of 5 or less indicates good sleep quality, whereas a score of 6 or more indicates poor sleep quality (16).

The Depression, Anxiety and Stress Scale (DASS) created by Lovibond and Lovibond (1996). is a well-established scale used in both clinical and experimental settings (19). In this study, the French short version, containing 21 items, was used (DASS-21). This version has reliable psychometric properties (20, 21). This questionnaire was designed to explore the negative emotional states of depression, anxiety and stress during the week preceding the day of assessment. Each dimension is evaluated using seven items, and each item is scored from 0 (does not apply to me at all) to 3 (applies to me very much or most of the time) (5, 22).

The global score of DASS-21 is classified into severity levels: “mild”, “moderate”, “severe” and “extremely severe” (19) (See **Table 1**).

**Table 1:** Interpretation of the DASS-21 score.

Subscale	Depression	Anxiety	Stress
Normal	0-9	0-7	0-14
Mild	10-13	8-9	15-18
Moderate	14-20	10-14	19-25
Severe	21-27	15-19	26-33
Very severe	28+	20+	34+

Exposure to ELF-EMFs was assessed by spot measurements taken over approximately a 2-minute period at fixed locations in the workplace, via a handheld measurement device according to IEEE Std C95.3.1 recommendations (23). For the EG, spot measurements were made in units B, C and D of the central power plant in Sousse city. For the NEG, the measurements were made in the administrative departments of the same company. Measurements of magnetic flux density, intensity (μT) and magnetic field strength (V/m) were made via an AKOZON portable EMFs tester available, from Amazon, France (24). This device has a measurement bandwidth of 5 Hz ~ 3500 MHz. It covers an electric field ranging from 1 V/m to 999 V/m and a magnetic field ranging from 0.01 μT to 99.99 μT with accuracies of 1 V/m and 0.01 μT. The alarm threshold is 40 V/m for the electric field and 0.4 μT for the magnetic field. In this study, 110 spot measurements were made, with 53 measurements for the EG and 57 measurements for the NEG.

### Statistical analysis

The data were analyzed via SPSS 23.0 software. Numerical variables were expressed as means and standard deviations, whereas categorical variables were expressed in terms of numbers and percentages.

The Student’s t-test was used to compare two means. Non-parametric tests (Mann-Whitney-U) were used for the ordinal data arising from the sub-scales of the PSQI and DASS-21. The chi-squared test was used to compare proportions. When the conditions of the chi-squared test were not met, Fisher’s exact test was used. The multivariate analysis was performed via binary logistic regression following a stepwise method.

Demographic and lifestyle variables such as university education and age were included as covariates in the regression in order to ascertain the effects that they may have on the main dependent variables (See **Table 2**).

**Table 2:** Sociodemographic characteristics of the exposed and non-exposed groups.

Variable	Category	EG n=77	NEG n=88	P value
Age (Years)	< 30	12 (15.6%)	10 (11.3%)	0.25*
	[30-40]	32 (41.5%)	29 (33%)	
	>40	33 (42.9%)	49(55.7%)	
University level	Yes	34 (44.2%)	50 (56.8%)	0.1**
	No	42 (55.8%)	38 (43.2%)	
Smoking	Yes	38 (49.4%)	40 (45.5%)	0.62**
	No	39 (50.6%)	48 (54.5%)	
Physical activity	Yes	53 (68.8%)	68 (77.3%)	0.22**
	No	24 (31.2%)	20 (22.7%)	
Obesity	Yes	19 (24.7%)	22 (25%)	0.77**
	No	58 (75.3%)	66 (75%)	

*Kruskal Wallis.

** Chi squared test.

All explanatory variables showing an association with the dependent variables (depressive symptoms, anxiety symptoms, and insomnia) at a significance level of p < 0.2 in univariate analyses were retained for inclusion in the initial multivariate regression models. Final models were derived through a backward elimination process, and the magnitude of associations between predictors and each outcome was expressed as adjusted odds ratios (aORs) with corresponding 95% confidence intervals (CIs). All the statistical tests were used at a level of significance of p < 0.05.

## Results

Age distribution was broadly similar between the EG and the NEG with predominance of participants aged over 40 years old, accounting for 42.9% and 55.7%, respectively (p=0.25). Sociodemographic characteristics were comparable between both groups (See **Table 2**).

The median value of job seniority was 9 years in the EG [min 2, max 35] and 12 years in the NEG [min 2, max 39], without significant differences (p =0. 11).

In the central power plant, high levels of ELF-EMF exposure were measured near the main power of the boiler in unit D (mean 40.34 μT). The results of the ELF-EMF measurements are summarized in **Table 3**.

**Table 3:** ELF-EMF measurements in the power plant and administrative departments.

TEGC unit	Spot measurements	Median (μT)	Minimum (μT)	Maximum (μT)	p
Power plant (B)	15	1.4	0.25	16	**<0.001**
Power plant (C)	17	4.84	0.1	23.5	
Power plant (D)	17	6.67	0.3	40.34	
Administrative departments	57	0	0	1	

p: Kruskal-Wallis.

ELF-EMFs, Extremely low-frequency electromagnetic fields; TEGC, Tunisian Electricity and Gas Company.

Bold p-values (p< 0.05) and corresponding Ors indicate statistically significant results.

According to the PSQI global score, 64.9% of the EG had poor sleep quality (PSQI score >5) compared to 29.5% of the NEG. The difference between the two groups was statistically significant (p<0.001). Compared with the NEG, the EG was four times more likely to have poor sleep quality (OR = 4.41; 95% CI [2.29-8.5]) (See **Table 4**).

**Table 4:** Distribution of exposed group and non-exposed group according to the interpretation of the PSQI score.

Variable Sleep quality exposure to ELF-EMFs	Poor (PSQI score >5)		Good (PSQI Score ≤5)		p	OR [95% CI]
	Number	Percentage (%)	Number	Percentage (%)		
Yes	50	**64,9**	27	35,1	<0.001^*^	**4,41** [2,29-8,5]
No	26	29,5	62	**70,5**		

*Chi squared test.

Bold p-values (p< 0.05) and corresponding Ors indicate statistically significant results.

According to the interpretation of the DASS-21 score, depression was observed in 24.7% of the EG participants but only in 3.4% of the NEG participants [(p<0.001) (OR = 9.28 95% CI [2.62-32.8]) (See **Table 5**). For the EG, depression was mild in 15.6% of cases, moderate in 5.2% of cases and extremely severe in 3.9% of cases (See **Table 6**). Anxiety, was reported in 23.4% of the EG participants and none of the NEG participants with a statistically significant difference (p<0.001). Anxiety was moderate in most cases (10.4%) (See **Table 7**). Stress was found in 46.7% of the EG and in none of the NEG. It was mild in most cases (27.3%) (See **Table 8**).

**Table 5:** Distribution of exposed group and non-exposed group according to the interpretation of the DASS-21 score for depression.

Variable Depression exposure to ELF-EMFs	Yes (DASS-21 score > 9)		No (DASS-21 score ≤ 9)		p	OR [95% CI]
	Number	Percentage (%)	Number	Percentage (%)		
Yes	19	24.7	58	75,3	**<0.001**	**9,28** **[2,62-32,8]**
No	3	3.4	85	96,6		

* Chi squared test.

Bold p-values (p< 0.05) and corresponding Ors indicate statistically significant results.

**Table 6:** Distribution of exposed group (EG) and non-exposed group (NEG) according to the classification of depression.

Interpretation of the DASS-21 score for depression	EG		NEG		p
	Number	Percentage (%)	Number	Percentage (%)	
A normal score (0-9)	58	75,3	85	96,6	*
Mild depression (10-13)	12	15,6	2	2,3	
Moderate depression (14-20)	4	5,2	1	1,1	
Severe depression (21-27)	0	0	0	0	
Very severe depression (28+)	3	3,9	0	0	
Total	77	100	88	100	

*Non applicable.

**Table 7:** Distribution of exposed group (EG) and non-exposed group (NEG) according to the interpretation of the DASS-21 score for anxiety.

Interpretation of the DASS-21 score for anxiety	EG		NEG		p
	Number	Percentage (%)	Number	Percentage (%)	
A normal score (0-7)	59	76,6	88	100	*
Mild anxiety (8-9)	5	6,5	0	0	
Moderate anxiety (10-14)	8	10,4	0	0	
Severe anxiety (15-19)	4	5,2	0	0	
Very severe anxiety (20+)	1	1,3	0	0	
Total	77	100	88	100	

*Non applicable.

**Table 8:** Distribution of exposed group (EG) and non-exposed group (NEG) according to the interpretation of the DASS-21 score for stress.

Interpretation of the DASS-21 score for stress	EG		NEG		p
	Number	Percentage (%)	Number	Percentage (%)	
A normal score (0-14)	41	53,2	88	100	*
Mild stress (15-18)	21	27,3	0	0	
Moderate stress (19-25)	14	18,2	0	0	
Severe stress (26-33)	1	1,3	0	0	
Very severe stress (34+)	0	0	0	0	
Total	77	100	88	100	

*Non applicable.

The components “subjective sleep quality”, “sleep disturbance”, “daytime dysfunction” and “sleep duration” presented higher scores in the EG compared to the NEG with a statistically significant difference. All the emotional states had a significant difference in the EG and NEG. The results of the PSQI and DASS-21 mean scores and their components are summarized in **Table 9**.

**Table 9:** PSQI and DASS-21 mean scores and their components.

Variable	EG		NEG		p*
	Median	Min–Max	Median	Min–Max	
Pittsburgh sleep quality index (PSQI)					
C1: Subjective sleep quality	1	0-3	1	0-3	**0.009**
C2: Sleep latency	1	0-3	1	0-3	0.13
C3: Sleep duration	1	0-3	1	0-3	**0.003**
C4:Habitual sleep efficiency	0	0-3	0	0-3	0.51
C5: Sleep disturbances	1	0-3	0,5	0-3	**0.02**
C6: Sleep medication use	0	0-3	0	0-2	0.32
C7: Daytime dysfunction	2	0-3	0	0-3	**<0.001**
Global Score of PSQI	6	1-16	4	0-15	**<0.001**
Depression, anxiety and stress scale (DASS-21)					
Depression	6.13 ± 6.41		1.25 ± 2.47		**<0.001**
Anxiety	4.68 ± 5.02		1.16 ± 1.68		**<0.001**
Stress	12.83 ± 6.82		4.02 ± 4.15		**<0.001**

*Mann–Whitney test.

Bold p-values (p< 0.05) and corresponding Ors indicate statistically significant results.

According to the bivariate analysis, the two factors that were significantly associated with poor sleep quality were university level (p=0.002; OR = 0.38, 95% CI [0.2–0.72]) and exposure to ELF-EMFs (p<0.001). Depression was significantly associated with physical activity as a protective factor (p=0.01; OR = 0.24; 95% CI [0.09–0.61]) and exposure to ELF-EMFs (p<0.001). A statistically significant association was noted between anxiety and university level as a protective factor (p=0.04; OR = 0.33; 95% CI [0.11–0.98]). Exposure to ELF-EMF was also significantly associated with anxiety (See **Table 10**).

**Table 10:** Factors significantly associated with sleep disorders, depression, anxiety and stress according to the bivariate analysis.

*Variables*	p	OR [95% CI]
*1)Sleep disorders according to PSQI score*		
University level	0.02	0.38 [0.2-0.72]
Exposure to ELF-EMFs	**<0.001**	--
*2)Depression according to DASS-21*		
Physical activity	0.01	0.24 [0.09-0.61]
Exposure to ELF-EMFs	**<0.001**	--
*3) Anxiety according to DASS-21*		
University level	0.04	0.33 [0.11-0.98]
Exposure to ELF-EMFs	**<0.001**	--
*4) Stress according to DASS-21*		
Being married	0.04	0.45 [0.2-0.99]
Job tenure	0.04	--
Exposure to ELF-EMFs	**<0.001**	--
Physical activity	0.02	0.3 [0.14-0.64]

OR, Odds ratio; ELF-EMFs, Extremely low-frequency electromagnetic fields.

After binary logistic regression, including the variables of interest, the factors that were independently associated with poor sleep quality were exposure to ELF-EMFs (p<0.001; aOR =1.28; 95% CI [1.14–1.43]) and university education (p=0.009; aOR=0.41; 95% CI [0.21–0.8]). Factors associated with depression were exposure to ELF-EMFs (p<0.001); aOR=1.45; 95% CI [1.17-1.81]) and physical activity (p=0.06: aOR =0.25; 95% CI [0.9-0.67]). After multivariate analysis, no factor was associated with anxiety. A statistically significant association was found between stress and age (p=0.003; aOR=0.95; 95% CI [0.89-0.98]) and stress and physical activity (p<0.001: aOR =0.22; 95% CI [0.07-0.66]) (See **Table 11**).

**Table 11:** Factors associated with poor sleep quality, depression and stress after multivariate analysis.

*Variables*	p	OR [95%CI]	p	aOR [95% CI]
Poor sleep quality (PSQI >5)				
University level	0.02	0.38 [0.2-0.72]	**0.009**	**0.41 [0.21-0.8]**
Exposure to ELF-EMFs	**<0.001**	--	**<0.001**	**1.28 [1.14-1.43]**
Depression				
Physical activity	**0.01**	**0.24 [0.09-0.61]**	**0.006**	**0.25 [0.9-0.67]**
Exposure to ELF-EMFs	**<0.001**	--	**<0.001**	**1.45 [1.17-1.81]**
Stress				
Age	0.09	**--**	**0.003**	**0.95 [0.89-0.98]**
Physical activity	**0.02**	**0.3 [0.14-0.64]**	**<0.001**	**0.22 [0.07-0.66]**

OR, Odds ratio; aOR, Adjusted odds ratio; ELF-EMFs, Extremely low-frequency electromagnetic fields.

Bold p-values (p< 0.05) and corresponding Ors indicate statistically significant results.

## Discussion

We conducted a cross-sectional study including 165 employees from the TEGC in order to assess the impact of ELF-EMFs on depression, anxiety, stress and sleep quality. To our knowledge, few epidemiological studies were conducted on this issue, which continues to draw the attention, not only, of the scientific community but also of the general public.

### Assessment of occupational exposure to ELF-EMFs

This study evaluated ELF-EMF exposures of workers in a power plant. The results revealed that the median value in the power plant was 5.86 μT [min0.1, max 40.34] with the maximal value recorded near the main boiler feeder pump. Values measured were below those recommended by the guidelines of the International Commission on Non-Ionizing Radiation Protection (ICNIRP), which vary between 100 and 1000 μT depending on the frequency range of the radiation (25, 26). Our results were similar to those reported in the literature. In a study evaluating the association between sleep quality and occupational exposure to electromagnetic fields in the petrochemical industry, the values measured ranged from 0.14 to 49.9 μT (27). Most values measured in epidemiological studies of occupational exposure to ELF-EMFs remain below the guideline values set by various organizations (28, 29). Nevertheless, the negative effects of ELF-EMFs reported in the literature may appear at levels below international safety limits (4, 30, 31).

### The impact of ELF-EMFs on sleep quality

In the present study, a significantly higher proportion of power plant workers reported poor sleep quality compared with administrative workers. Moreover, the multivariate analysis showed that ELF-EMFs and university level were significantly associated with sleep quality.

In the study of Hosseinabadi et al. (5) assessing the impact of ELF-EMF exposure in a power plant on sleep quality, the results indicated that the global PSQI score was 7.41 ± 3.89 for the EG and 6.02 ± 3.03 for the NEG. The main differences were especially noticeable in the “sleep efficiency” and “subjective quality of sleep” components.

An experimental study enrolled 18 healthy subjects who were good sleepers. These subjects spent a night sleeping in a wooden cabin and were blindly exposed to 1 μT of EMFs generated by a 50 Hz alternating current. EMFs, light and noise from other sources were monitored. The results revealed that exposure to EMFs disrupted sleep quality, with statistically significant reductions in sleep duration, sleep efficiency and slow-wave sleep (32). Another experimental study showed a direct association between exposure to EMFs and disturbances in sleep quality. While healthy volunteers were exposed to intermittent EMFs during a night’s sleep, significant changes in electroencephalogram (EEG) parameters were observed. These changes included a reduction in total sleep duration, a decrease in sleep efficiency, an increase in the duration of stage II sleep and a decrease in the duration of rapid eye movement (REM) sleep (33).

In another study, the effect of occupational exposure to ELF-EMFs on the mental health of employees in an electrolysis unit was assessed via the GHQ-28 (General Health Questionnaire). The results revealed significant differences between the EG and NEG in terms of GHQ-28 scores and self-reported symptoms of depression, anxiety and sleep disorders (11).

The main mechanism that was suggested to explain the impact of ELF-EMFs on sleep quality is the reduction of melatonin secretion by the pineal gland. In a cross-sectional study carried out among electronic equipment repairers, the results showed that these workers had a lower level of serum melatonin than the controls. The authors concluded that electronic equipment repairers, who are daily exposed to ELF-EMFs, are at high risk of oxidative stress which can be explained by a low level of melatonin (15).

However, the impact of ELF-EMFs on melatonin is not well established since the majority of studies yielded contradictory and negative results on humans (34, 35). The research on melatonin and ELF-EMFs has shifted recently, into its role as radio-protective agent. This hormone, by its free radical scavenging properties, has been reported to protect cells against oxidative stress induced by ELF-EMFs (36).

### The impact of ELF-EMFs on depression, anxiety and stress

Our study revealed that depression was more frequent among the power plant workers than the administration workers. Besides, a significant association was observed between depression and physical activity, which is considered a protective factor.

Our results were consistent with those of Hosseinabadi et al. (5), who reported a significant difference in the DASS-21 score for depression between the EG and the NEG (p=0.039). Depression was more common in the EG with a linear increase in the DASS-21 score as the level of ELF-EMFs increased. Another study conducted by Ghotbi et al. (11) indicated that symptoms of depression (p=0.007), anxiety and sleep disorders (p=0.001) were more common in exposed subjects working in an electrolysis unit.

In a study evaluating the psychological effects of occupational exposure to ELF-EMFs among 103 workers at high-voltage power stations, the authors assessed mental health via the SCL90-R Symptom Checklist. A higher prevalence of symptoms of depression, paranoia, psychosis and anxiety was observed in the exposed subjects (37).

The association between exposure to EMFs and suicide was studied by Van Wijngaarden et al. (38) among workers in the electricity sector. The authors found that mortality by suicide was greater in exposed subjects and that the risk increased with the duration of exposure. The mechanism suggested by the authors was the disruption of melatonin production by EMFs, which plays an important role in depression.

Our results showed that anxiety was more prevalent in the EG than in the NEG. After multivariate analysis, no factor was significantly associated with anxiety.

Similar results were reported in an Iranian study evaluating the effects of occupational exposure to ELF-EMFs in power plant workers. The results revealed a mean DASS-21 anxiety score of 12.61 ± 4.99 for the EG and 12.24 ± 3.49 for the NEG, with no statistically significant difference. However, a linear increase in anxiety level was noted as the level of exposure to ELF-EMFs increased (5).

Karimi et al. (39) conducted an experimental study on the effects of ELF-EMFs on memory, anxiety and oxidative stress. The rats were exposed to EMFs for 60 days and then anxiety was assessed. The results revealed that exposure to EMFs increased the level of anxiety and the concentrations of several biological markers of oxidative stress, particularly malondialdehyde (MDA). Another experimental study conducted by Balassa et al. (40) evaluated the impact of continuous exposure to 50 Hz and 500 μT EMFs for 21 days on the behavior of rats. The results revealed a significant increase in stress- and anxiety-related behaviors.

It has been suggested that chronic exposure to ELF-EMFs can cause significant changes in behavior by increasing anxiety and depression. These two effects are strongly linked to chronic stress (41). In fact, exposure to ELF-EMFs in animal studies using rats is considered to be a moderate stress situation that activates a wide spectrum of neuronal, molecular and neurochemical reactions leading to physiological and behavioral responses (42, 43).

One of the mechanisms that may explain the effects of EMFs on mental health is oxidative stress (44, 45). In 2008, an animal study assessing the impact of continuous exposure to EMFs for 30 days on certain biological parameters in mice revealed a significant decrease in glucose levels and alkaline phosphatase activity. On the other hand, lactate dehydrogenase and glutathione transferase activities increased significantly. The authors concluded that there is a link between exposure to ELF-EMFs and oxidative stress by disturbing the redox balance (46).

### Study limitations

Finally, study limitations should be considered. First, the relatively small sample size limits the statistical power and the generalizability of findings. Second, a potential selection bias may arise from the “healthy worker effect”, because employees on sick leave or deemed unfit for work were excluded. However, this would have led to an underestimation of the prevalence of sleep disturbances, depression, anxiety, and stress.

Furthermore, the cross-sectional design does not allow the establishment of causal relationships between ELF-EMF exposure and health outcomes. The use of self-reported questionnaires to assess psychological and sleep-related symptoms introduces the possibility of subjective reporting bias, although previous studies suggest moderate concordance between self-reported and objective sleep data.

Despite these limitations, our study benefits from a relatively homogeneous population in terms of socioeconomic characteristics, lifestyle habits, and occupational environment, as all participants belonged to the same company and shared similar working conditions. Data collection was standardized and conducted by a single physician, minimizing measurement bias. Additionally, ELF-EMF exposure was objectively assessed through multiple spot measurements across various workplace locations, enhancing exposure characterization.

## Conclusion

Our study revealed that occupational exposure to ELF-EMFs may negatively affect sleep quality and mental health, even at levels far below regulatory guidelines for this type of fields. These findings underscore the importance of implementing safer workplace practices and incorporating them into Occupational Health and Safety training.

## Data Availability

The raw data supporting the conclusions of this article will be made available by the authors, without undue reservation.
